# Characterization of avian *LEAP2* transcripts with alternative polyadenylation and their 3′UTR stability

**DOI:** 10.1016/j.psj.2026.108027

**Published:** 2026-10-21

**Authors:** Hui Wang, Xin Shu, Yutong Wang, Bingjie Xu, Li Liu, Yimeng Li, Ziqiu Niu, Xiaotong Zheng, Jianfei Chen

**Affiliations:** a Jiangsu Key Laboratory of Sericultural and Animal Biotechnology, School of Biotechnology, Jiangsu University of Science and Technology, Zhenjiang, 212100, China; b Key Laboratory of Silkworm and Mulberry Genetic Improvement, Ministry of Agriculture and Rural Affairs, Sericultural Scientific Research Center, Chinese Academy of Agricultural Sciences, Zhenjiang, 212100, China

**Keywords:** *LEAP2*, UTR, Gene expression profile, Gene stability, RNA binding protein, miRNA

## Abstract

LEAP2 is a key regulator of appetite and metabolism, yet its transcriptional regulation, especially alternative polyadenylation (**APA**)‑derived transcript isoforms under different feeding states, remains largely uncharacterized in poultry. Here, combining tissue expression profiling, 5′/3′‑RACE, and dual‑luciferase reporter assays, we systematically characterized *LEAP2* transcript isoforms in avian species and mice. Tissue profiling showed duck *LEAP2* was highly expressed in liver, kidney and small intestine, whereas mouse *Leap2* was mainly restricted to liver and small intestine. Two APA generated *LEAP2* isoforms with identical coding sequences but distinct 3′UTR lengths (the long and short variants designated **l‑3′UTR** and **s‑3′UTR**, respectively) were identified in all tested avian species, and homologous isoforms existed in mice; the avian l‑3′UTR sequence was highly conserved across poultry species but divergent from the mouse ortholog. Feeding-status assays revealed post-prandial alterations in avian l-3′UTR-*LEAP2* abundance. After fasting-refeeding, the l‑3′UTR isoform predominated in ducks (two‑thirds of total transcripts), whereas mouse *Leap2* was unresponsive to feeding. Dual-luciferase assays demonstrated species-dependent 3′UTR stability. The s-3′UTR was more stable in chicken and quail, while l-3′UTR was more stable in ducks. Mechanistically, RNA-binding proteins (**RBPs**) PUM2 and ZFP36 were validated to modulate l‑3′UTR stability, and two miRNAs (gga‑miR‑2188‑3p, gga‑miR‑190a‑3p) were confirmed to target the 3′UTR and regulate *LEAP2* expression in hepatocytes. Collectively, this study identifies conserved APA‑derived *LEAP2* 3′UTR isoforms in poultry with species‑specific stability co‑regulated by RBPs and miRNAs, laying a basis for further mechanistic investigation of *LEAP2* in avian energy homeostasis.

## Introduction

Liver enriched antimicrobial peptide 2 (**LEAP2)** was originally cloned and identified as an antimicrobial peptide in mammals and poultry (Krause et al., 2003; Lynn et al., 2004; Townes et al., 2004; Ishige et al., 2016), and its antibacterial activity has long been the primary focus of related investigations. In mammals, LEAP2 kills Gram-positive bacteria such as Bacillus by disrupting bacterial membrane integrity or binding microbial DNA (Hocquellet et al., 2010). Its expression is dysregulated under multiple infectious and inflammatory conditions. Colonic *LEAP2* mRNA is significantly downregulated in HIV/HCV co-infected patients and negatively correlated with the immune activation marker sCD27 (Shata et al., 2013). *LEAP2* is detectable in human palatine tonsils (Ball et al., 2007), and tonsillar *LEAP2* is markedly suppressed in patients with allergic rhinitis (Bogefors et al., 2014). By contrast, cerebrospinal fluid LEAP2 concentrations are elevated in bacterial meningitis patients, supporting its potential as a diagnostic biomarker (Sakai et al., 2021). Poultry studies further demonstrate that *LEAP2* expression responds rapidly to pathogenic infection. *Salmonella enterica* infection upregulates intestinal and hepatic *LEAP2* by 2–3 fold to directly kill bacteria via outer membrane damage (Townes et al., 2004; Shao et al., 2016). In healthy chickens, *LEAP2* exhibits tissue-specific constitutive high expression in the liver, intestine and gonads; *Salmonella enteritidis* infection further elevates gonadal *LEAP2* in 28-week-old roosters and hens (Michailidis, 2010). *Campylobacter* infection specifically boosts cecal *LEAP2* without altering expression in other intestinal segments. *Eimeria* infection downregulates intestinal *LEAP2* mRNA by 50% in broilers, linked to villus atrophy and depleted goblet cells (Su et al., 2015; Su et al., 2017; Cloft et al., 2023), while *Eimeria maxima* induces robust splenic *LEAP2* upregulation in Cobb broilers (Froebel et al., 2024). *Clostridium perfringens* infection markedly suppresses jejunal *LEAP2* expression in broilers (Goo et al., 2024). Genetic selection also shapes baseline *LEAP2* levels: Fayoumi disease-resistant chickens show 1.8-fold higher basal bone marrow *LEAP2* expression than Leghorns, with stronger induction upon poly(I:C) viral mimic stimulation (Jang et al., 2020). Collectively, these findings validate the critical roles of *LEAP2* in avian mucosal immunity and disease-resistant breeding, consistent with the consensus that earlier LEAP2 research predominantly addressed its antibacterial functions.

Several studies have dissected the structural features and antibacterial mechanisms of LEAP2 and their linkage to disulfide bonds, and proposed that antimicrobial activity may not represent its core physiological function, implying the existence of uncharacterized biological roles (Henriques et al., 2010; Hocquellet et al., 2010). In 2018, Ge et al. reported a landmark discovery demonstrating that LEAP2 acts as an endogenous appetite-regulating hormone (Ge et al., 2018), which has directed subsequent investigations into its anorexigenic effects (Shankar et al., 2021; Hagemann et al., 2022). LEAP2 serves as the natural ligand of growth hormone secretagogue receptor (**GHSR**), and this interaction is highly conserved across vertebrates; the first 14 amino acids of mature LEAP2 peptide are indispensable for GHSR binding (M Kadmi et al., 2019; Wang et al., 2019). Functionally, LEAP2 acts as an endogenous antagonist and inverse agonist of GHSR to counteract ghrelin-mediated signaling (Mustafá et al., 2021; Shankar et al., 2021; Fernandez et al., 2022). Clinical evidence confirms that LEAP2 participates in the regulation of metabolic disorders including obesity, diabetes, fatty liver and hepatic inflammation (Shankar et al., 2024; Miguéns et al., 2026), with numerous clinical reports linking LEAP2 to body mass index, appetite control, energy homeostasis and immune mediator secretion. Consistent with mammalian observations, avian studies reveal robust hepatic and intestinal *LEAP2* expression in chickens and quails, whose transcript abundance is tightly coupled to feeding status, with fasting significantly suppressing *LEAP2* expression (Zheng et al., 2022; Shu et al., 2024). Both intraperitoneal delivery of the 14‑amino‑acid mature peptide and intracerebroventricular administration of mature chicken LEAP2 peptide markedly reduce feed intake in young chicks, as previously described (Saffar et al., 2025; Chen et al., 2026). Moreover, intestinal *LEAP2* expression is higher in high-body-weight broilers relative to low-body-weight individuals (Kinstler et al., 2024). These lines of evidence identify LEAP2 as a key modulator of appetite and energy metabolism in poultry.

Overall, previous mammalian and avian studies of LEAP2 have mainly focused on its expression patterns and dual functions in immunity and metabolic homeostasis. Research on *LEAP2* transcript variants remains limited, and the few published reports have almost exclusively described intron‑retention alternative splicing events rather than alternative polyadenylation (**APA**)‑derived 3′UTR length variants (Krause et al., 2003; Townes et al., 2004; Howard et al., 2010; Sosinski et al., 2025). Our prior quail study identified a novel feeding-induced long 3′UTR *LEAP2* isoform (Shu et al., 2024), whose function, regulation and conservation remain uncharacterized across vertebrates. Given that APA‑mediated 3′UTR diversity is a core post‑transcriptional mechanism tuning mRNA stability and nutritional responsiveness (Mayr, 2016; Xu et al., 2026), its role in LEAP2 feeding regulation represents a key gap in understanding avian energy homeostasis. We hypothesize that *LEAP2* APA-derived long and short transcript isoforms (**l-*LEAP2*, s-*LEAP2***) are conserved across species, display species-specific differences in mRNA stability, and are co-regulated by regulatory factors including RNA-binding proteins (**RBPs**) and miRNAs to mediate feeding responsiveness. To test these hypotheses, we cloned full-length *LEAP2* from mice, chickens and ducks, and characterized avian 3′UTR isoform stability and its regulatory determinants. This work lays a foundation for studying *LEAP2* transcriptional regulation.

## Materials and methods

### Animals and sample collection

The animal protocols were approved by the Institutional Animal Care and Use Committee (IACUC) of Jiangsu University of Science and Technology (G2025SJ16 for mouse, GQ20230302 and S2022SJ13 for chicken and duck, Zhenjiang, China). Animal care and handling practices were followed by the IACUC guidelines.

Beijing ducks (*Anas platyrhynchos*) and Kunming outbred mice (*Mus musculus*) were obtained from a licensed commercial supplier, where all routine vaccinations and standard health management had been completed prior to delivery. Upon arrival, animals were acclimatized under standardized husbandry conditions (25 ± 1°C, 50–60% relative humidity, 12h light/dark cycle) with ad libitum access to basal diet and clean water. In total, 12 ten‑day‑old male ducks and 12 37‑day‑old male mice with comparable body weights within each species were selected for gene‑expression profiling and 5′/3′‑RACE assays. Animals of each species were randomly assigned to three groups (n = 4 per group): *ad libitum* fed control, 18‑h‑fasted, and refed (18 h fasting followed by 2 h re‑feeding). No additional medications or vaccinations were administered during the entire experimental period to avoid confounding effects. Animals were euthanized via CO₂ asphyxiation. Tissue samples including brain, heart, liver, spleen, lung, kidney, proventriculus (chicken), gizzard (chicken), mouse stomach, duodenum, jejunum, ileum, caecum, colon, skin and pectoralis muscle were immediately frozen in liquid nitrogen and stored at −80 °C for subsequent RNA isolation. Chicken samples (15‑day‑old and 42‑day‑old) were collected from Arbor Acres broilers (*Gallus gallus*) described previously (Zheng et al., 2022; Chen et al., 2026). Liver and duodenum tissues from chickens, ducks and mice were subjected to rapid amplification of cDNA ends (**RACE**), whereas all collected tissue samples were used for gene‑expression profiling. Fertile Yao chicken eggs were obtained from commercial markets and incubated in an automatic incubator (Ruitai Incubation Equipment Development Center, Dezhou, China) under standardized hatching conditions (37.8°C and 60% relative humidity). Hepatocytes and intestinal epithelial cells were separately isolated and cultured from chicken embryos at embryonic day 14 (**E14**) and day 19 (**E19**), respectively.

### Total RNA extraction

Total RNA was extracted from each tissue sample using the RNAiso Plus regent (Takara Biotech Co., Ltd, Beijing, China) as per the manufacturer’s protocol. Agarose gel electrophoresis was applied to assess RNA integrity, while a Nanophotometer N60 Touch instrument (IMPLEN, Munich, Germany) was used to determine RNA concentration. Purified total RNA aliquots were preserved at −80 °C for subsequent experiments.

### Cloning the full-length cDNA of the LEAP2

The 5′/3′-RACE assay for the *LEAP2* gene was performed using the HiScript‑TS 5′/3′‑RACE Kit (Vazyme Biotech Co., Ltd, Nanjing, China) according to the manufacturer’s instructions. RACE primers were designed against the *LEAP2* CDS sequences retrieved from the NCBI database. Nested gene‑specific primers were employed when the primary PCR failed to yield desirable amplicons. RACE primers for quail were adopted from a previous study (Shu et al., 2024). All RACE primers are listed in Table S1. DNA Marker 2000 (ShengGong Biotech, Shanghai, China; or Sevier Biological Technology Co. Ltd., Wuhan, China) was used to estimate the size of PCR products. The PCR amplicons were subcloned with the Ultra‑Universal TOPO Cloning Kit (Vazyme), followed by Sanger sequencing (Shangya Biotechnology Co., Ltd., Hangzhou, China).

### cDNA synthesis, RT-PCR amplification, and RT-qPCR analysis

First-strand cDNA synthesis was performed using 1 μg of total RNA and the HiScript III 1st Strand cDNA Synthesis Kit with gDNA wiper (Accurate Biotechnology Co., Ltd., Changsha, China). The resulting cDNA products were diluted at a 1:3 ratio and stored at −20°C for subsequent RT-PCR and RT-qPCR detection. All primers used for RT-qPCR and RT-PCR amplification were designed via Primer Premier 5.0. Detailed primer sequences are summarized in Table S2 and Table S3, and all primers were synthesized by Shangya Biotechnology. RT-qPCR assays were carried out using the SYBR Green Premix Pro Taq HS qPCR Tracking Kit II (Accurate Biotechnology). Each sample was tested in three technical replicates. The RT‑qPCR reaction system and operational procedures were performed as previously described, whereas TBP and RPL13 served as reference genes for normalization, as reported previously (Chen et al., 2023). For RT-PCR detection, the reaction system and experimental protocols were strictly performed in accordance with the manufacturer’s guidelines of the 2 × Taq Plus Master Mix (Vazyme). Finally, the amplified PCR products were separated by 1% agarose gel electrophoresis and visualized for observation.

### Vector construction and oligonucleotides

The 3′UTR sequences of the *LEAP2* gene from different avian species were amplified using Phanta UniFi Master Mix (Vazyme). Primer details are provided in Table S4. Site‑directed mutagenesis of specific regions within the 3′UTR was achieved via splicing‑by‑overlap‑extension PCR. Primers for mutagenesis and the sequences of mutated 3′UTR fragments are summarized in Table S4 and Table S5. Following Sanger sequencing (Shangya Biotechnology), the pmir‑GLO reporter plasmids carrying wild‑type or mutated 3′UTR sequences were assembled by homologous recombination with 2 × Ezmax Universal CloneMix (Tolo Biotech, Shanghai, China). miRNA mimics and their corresponding inhibitors were chemically synthesized by GenePharma Co., Ltd. (Suzhou, China), and their sequences are shown in Table S6.

### Cell isolation and culture

Primary chicken embryonic hepatocytes were isolated from E14 embryos using an optimized protocol modified from a previous report (Shu et al., 2025a). Dissected liver tissues were rinsed 2–3 times with sterile PBS and minced into 1–2 mm³ fragments. Tissues were digested with 1% trypsin at 37°C for 15 min, and the resulting cell suspension was sequentially filtered through 100 μm, 70 μm, and 40 μm strainers to collect purified hepatocytes. Cells were cultured in DMEM (Gibco, Shanghai, China) supplemented with 1% penicillin/streptomycin, under standard incubation conditions (37°C, 5% CO_2_) Primary chicken intestinal epithelial cells were harvested from E19 embryonic intestines via a modified method (Shu et al., 2025b). Isolated intestinal tissues were washed thoroughly with sterile PBS and cut into 1–2 mm³ pieces. Tissues were digested in DMEM/F12 medium containing 1 mg/mL type I collagenase at 37°C for 30 min, with shaking every 5 min during digestion. The reaction was terminated with 10% fetal bovine serum-containing DMEM/F12. After gentle pipetting, the suspension was filtered through sterile gauze and centrifuged at 800 rpm. The cell pellet was washed with PBS and re-centrifuged for purification. Final cells were cultured in complete DMEM/F12 (Gibco) medium containing 10% premium fetal bovine serum (Wuhan Pricella Biotechnology Co., Ltd, China) and 1% penicillin/streptomycin, and incubated at 37°C with 5% CO_2_.

### Transfections, and dual luciferase assay

When cells reached 70%–80% confluency, they were subjected to transfection. Vector transfection was carried out using ExFect Transfection Reagent (Vazyme) according to the manufacturer’s instructions. 24h after transfection with plasmids encoding the 3′UTR sequences of l- and s-*LEAP2* transcripts as well as mutant constructs, the culture medium was refreshed, and 5 μM actinomycin D (Alading, Shanghai, China) was supplemented. Following treatment for 4 h, 8 h, and 12 h, cells were lysed, and Firefly and Renilla luciferase substrates were added using the Dual Luciferase Reporter Assay Kit (Vazyme) in accordance with the manufacturer’s protocols. Firefly luciferase activity was normalized to Renilla luciferase activity. Oligonucleotide transient transfection was performed with siRNA‑Mate Plus (GenePharma) following the manufacturer’s guidelines. Biological experiments were performed in three independent replicates, and each assay contained three technical replicates. Cells were harvested 40 h post‑transfection, and total RNA was extracted for RT‑qPCR quantification of *LEAP2*.

### Bioinformatics

Sequence alignment was performed using DNAMAN V6. Prediction of RBP binding sites within the 3′UTR sequences of *LEAP2* from chicken, quail, duck, and mouse was carried out via the RBPDB database (http://rbpdb.ccbr.utoronto.ca/). Since miRNA‑binding datasets for duck and quail were not available in this database, the chicken miRNA dataset was adopted for predictions for these two avian species. APA sites of the chicken *LEAP2* gene were predicted using the online resource Animal‑APAdb (https://gong_lab.hzau.edu.cn/Animal‑APAdb/).

### Statistical analysis

The relative mRNA expression was calculated using the 2^−△CT^ method. The individual with the lowest *LEAP2* expression in the ileum of the fed group was set to 1, and expression values of all other tissues were presented relative to this individual. The 2^–△△CT^ method was also applied for *LEAP2* quantification in cultured cells. Two reference genes were used for expression normalization; their geometric mean was taken as the final expression value, following a previously reported strategy for tissue‑expression profiling (Zheng et al., 2022). One-way ANOVA with a fixed-effect model (y_ij_ = μ + τ_i_ +ε_ij_) was used to evaluate statistical differences among groups. Residual normality was examined by Shapiro-Wilk test, and homogeneity of variances was assessed using Brown–Forsythe test in GraphPad Prism 10.1.2. Tukey’s post-hoc test was performed for pairwise comparisons. *P* < 0.05 was regarded as the threshold for statistical significance.

## Results

### Full-length cDNA cloning of LEAP2 in different species

To characterize the predominant expression tissues of the *LEAP2* gene in ducks and mice, we constructed tissue expression profiles of *LEAP2* using RT-qPCR. The results revealed that duck *LEAP2* was highly expressed in the liver, kidney, duodenum, jejunum and ileum, with relatively low expression levels detected in other tissues (Fig. 1A). In mice, *LEAP2* exhibited high expression mainly in the liver, duodenum, jejunum and ileum (Fig. 1B). Accordingly, pooled liver and intestinal RNA from each species was subjected to 5′/3′-RACE for *LEAP2*. A single dominant band was observed in the 5′-RACE gel for mice, chickens, quails and ducks (Fig. 2A). Two major bands were detected in the 3′-RACE gel of chickens, quails and ducks, whereas three bands were found in mice (Fig. 2B). Subsequent Sanger sequencing confirmed that *LEAP2* generates two mRNA isoforms with distinct 3′UTR lengths across all examined species. Full-length cDNA sequences of chicken *LEAP2*, duck *LEAP2* and mouse *Leap2* genes were successfully cloned using 5′/3′-RACE. The sequence of the long mouse *Leap2* transcript matched the GenBank reference entry (Accession: NM_153069.3). The full-length *LEAP2* sequences from chickens and ducks exhibited nucleotide variations and differed in transcript boundaries; these sequences were deposited in GenBank under accession numbers PZ836229–PZ836233. The transcript isoforms within each species share identical CDS regions but differ in 3′UTR length. RT-PCR further validated the existence of the *LEAP2* transcript carrying the l-3′UTR (Fig. 2C).

**Fig. 1:**
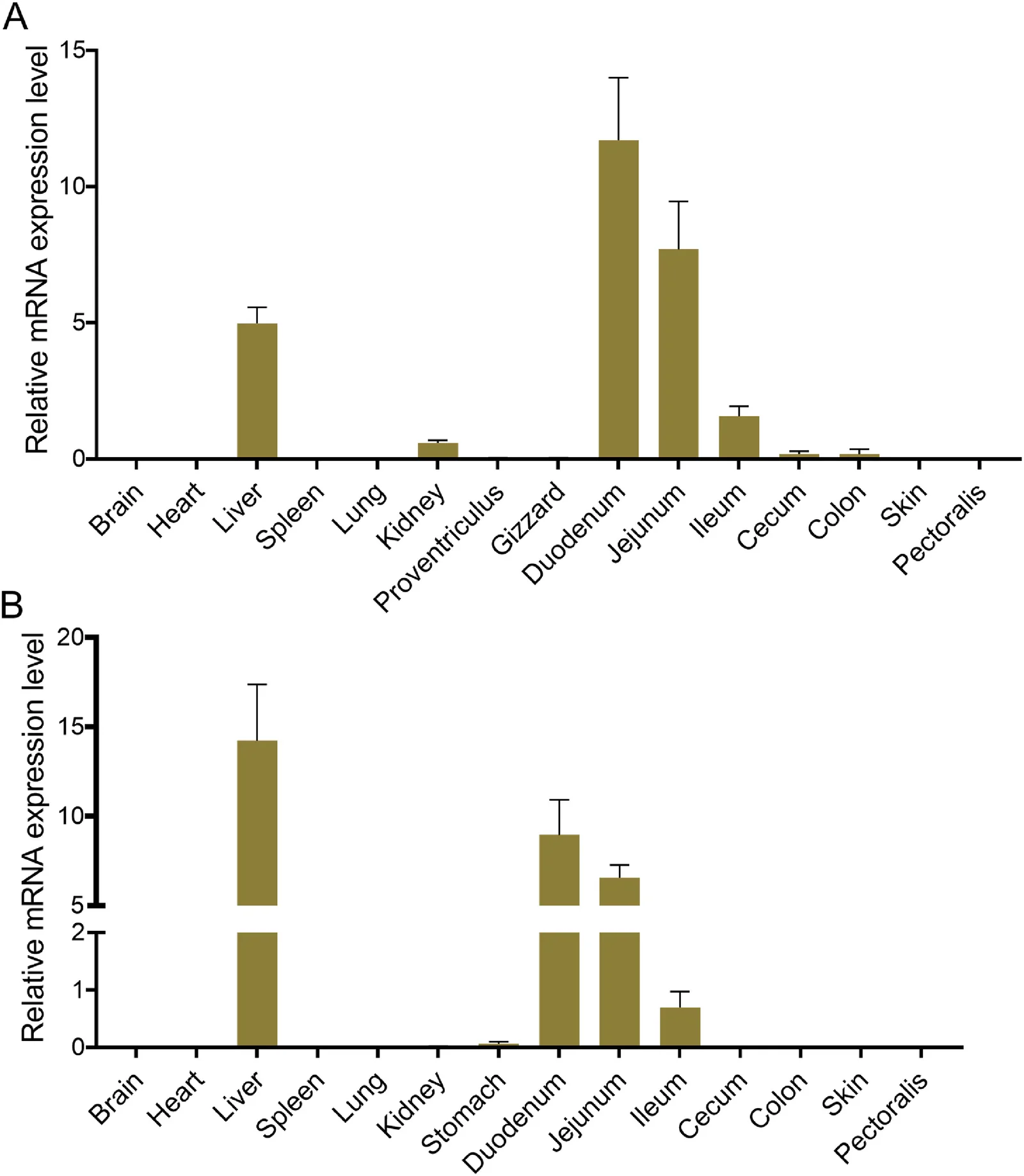
Relative expression profiles of *LEAP2* in multiple tested tissues of duck (A) and mouse (B).

**Fig. 2:**
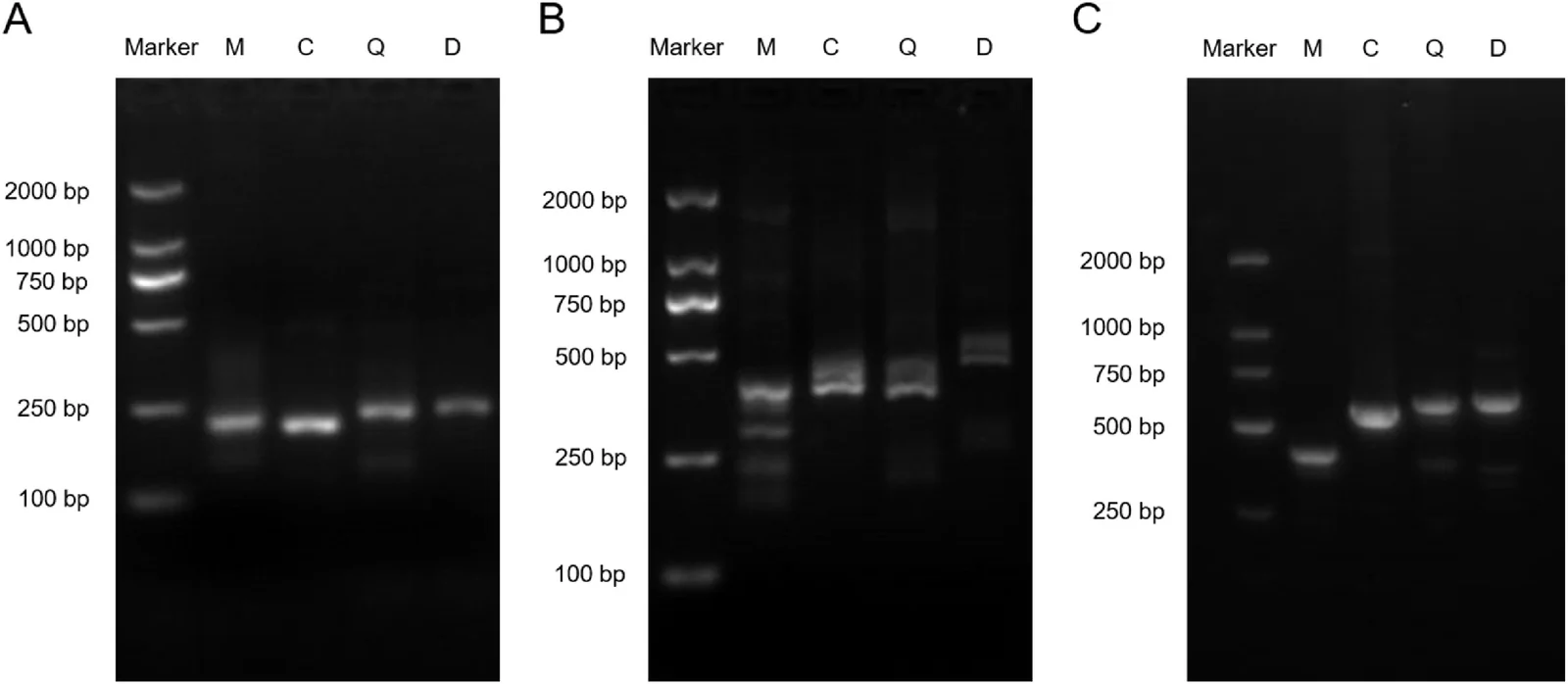
Agarose gel electrophoresis analysis of 5′and 3′-RACE products of the *LEAP2* gene from different species. (A) 5′-RACE. (B) 3′-RACE. (C) RT-PCR validation of long-*LEAP2* transcripts. M, C, Q, and D denote mouse, chicken, quail, and duck, respectively. The DNA Marker 2000 used in panels A and B was sourced from Sevier Biological Technology, whereas Marker 2000 in panel C was obtained from Sangon Biotech.

### LEAP2 isoform expression under distinct energy status

To investigate the association between l-*LEAP2* transcripts and energy metabolism across different species, RT-qPCR was performed to quantify the expression of total *LEAP2* (**t-*LEAP2***) and l-*LEAP2* transcripts under distinct feeding conditions in chickens, ducks, and mice. Tissue expression analysis in chickens showed that the hepatic, duodenal, and jejunal expression levels of t-*LEAP2* and l-*LEAP2* were significantly decreased in the fasted group compared with the fed group. No significant differences were observed in the refed group. In the ileum, the expression of t-*LEAP2* and l-*LEAP2* remained unchanged among all feeding groups (Fig. 3A). Moreover, 42 days old AA chickens exhibited tissue expression patterns of t-*LEAP2* and l-*LEAP2* similar to those of chicks (Fig. S1). In ducks, significantly lower expression levels of t-*LEAP2* and l-*LEAP2* were detected in the liver, kidney, duodenum, and jejunum under fasting conditions relative to the fed state, while no significant differences were observed in the refed group. In the ileum, the expression of t-*LEAP2* and l-*LEAP2* showed no significant difference between the fed and fasted groups, whereas the refed group differed significantly from both the fed and fasted groups. The proportion of l-*LEAP2* accounted for 2/3 of total *LEAP2* transcripts in the fasted and refed groups, whereas only approximately 30% in the fed group (Fig. 3B). In mice, the expression of t-*LEAP2* and l-*LEAP2* in the liver, kidney, duodenum, jejunum, and ileum showed no significant changes between the fed and fasted groups. Significant expression differences were only observed in the liver of the refed group, with no alterations detected in other tissues. Notably, l-*LEAP2* was the dominant transcript isoform in mice under all feeding conditions, occupying over 90% of total *LEAP2* transcripts (Fig. 3C).

**Fig. 3:**
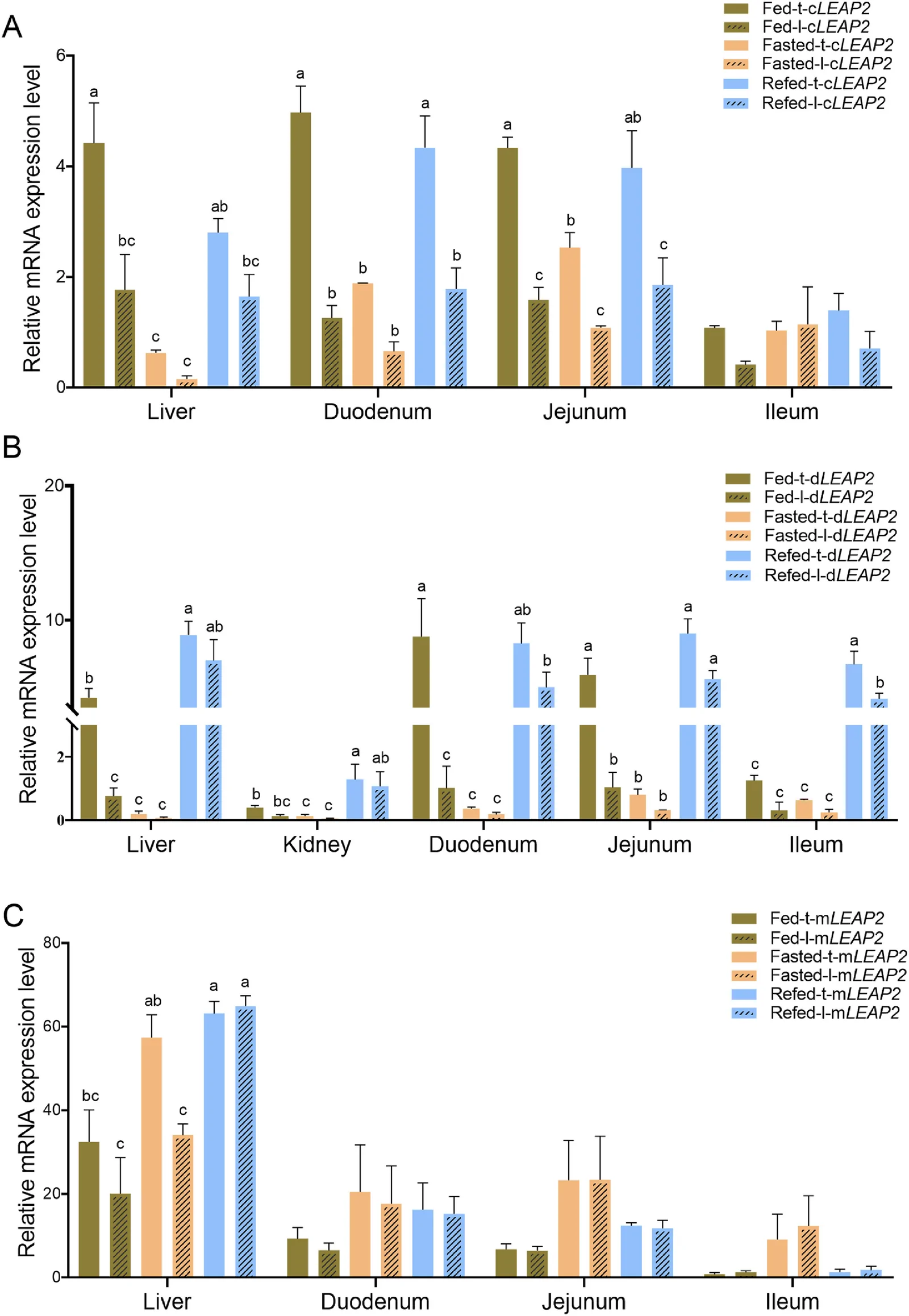
Relative expression of *LEAP2* isoforms in the main tissues of chicken (15 days) (A), duck (B) and mouse (C). The "t" and "l" in t-*LEAP2* and l-*LEAP2* stand for "total" and "long", respectively. Different lowercase letters denote significant differences within the same tissue (*P* < 0.05).

### Differential stability of 3′ UTRs between avian LEAP2 isoforms

Given that avian *LEAP2* generates long and short transcript isoforms with distinct proportional abundances across avian species, we next examined whether these isoforms differ in transcript stability. To explore their functions, pmir-GLO luciferase reporter vectors containing the 3′UTR sequences of l- and s-*LEAP2* transcripts from each species were constructed (Fig. 4A). Dual-luciferase reporter assays were subsequently performed using primary chicken embryonic hepatocytes and primary chicken embryonic intestinal cells. The luciferase activity was used to indirectly evaluate the stability of the corresponding 3′UTRs. The results revealed that the chicken *LEAP2* 3′UTR exhibited higher stability than those from duck and quail in primary chicken embryonic hepatocytes. Within each species, cs-3′UTR-*LEAP2* was more stable than cl-3′UTR-*LEAP2*, and qs-3′UTR-*LEAP2* was more stable than ql-3′UTR-*LEAP2*. Similarly, ds-3′UTR-*LEAP2* displayed greater stability compared with dl-3′UTR-*LEAP2* in ducks (Fig. 4B). The stability patterns of l- and s-3′UTRs across species in intestinal cells were consistent with those observed in hepatocytes. One notable difference was that qs-3′UTR-*LEAP2* showed stability comparable to cs-3′UTR-*LEAP2* in intestinal cells; both represented relatively stable UTR variants( Fig. 4C).

**Fig. 4:**
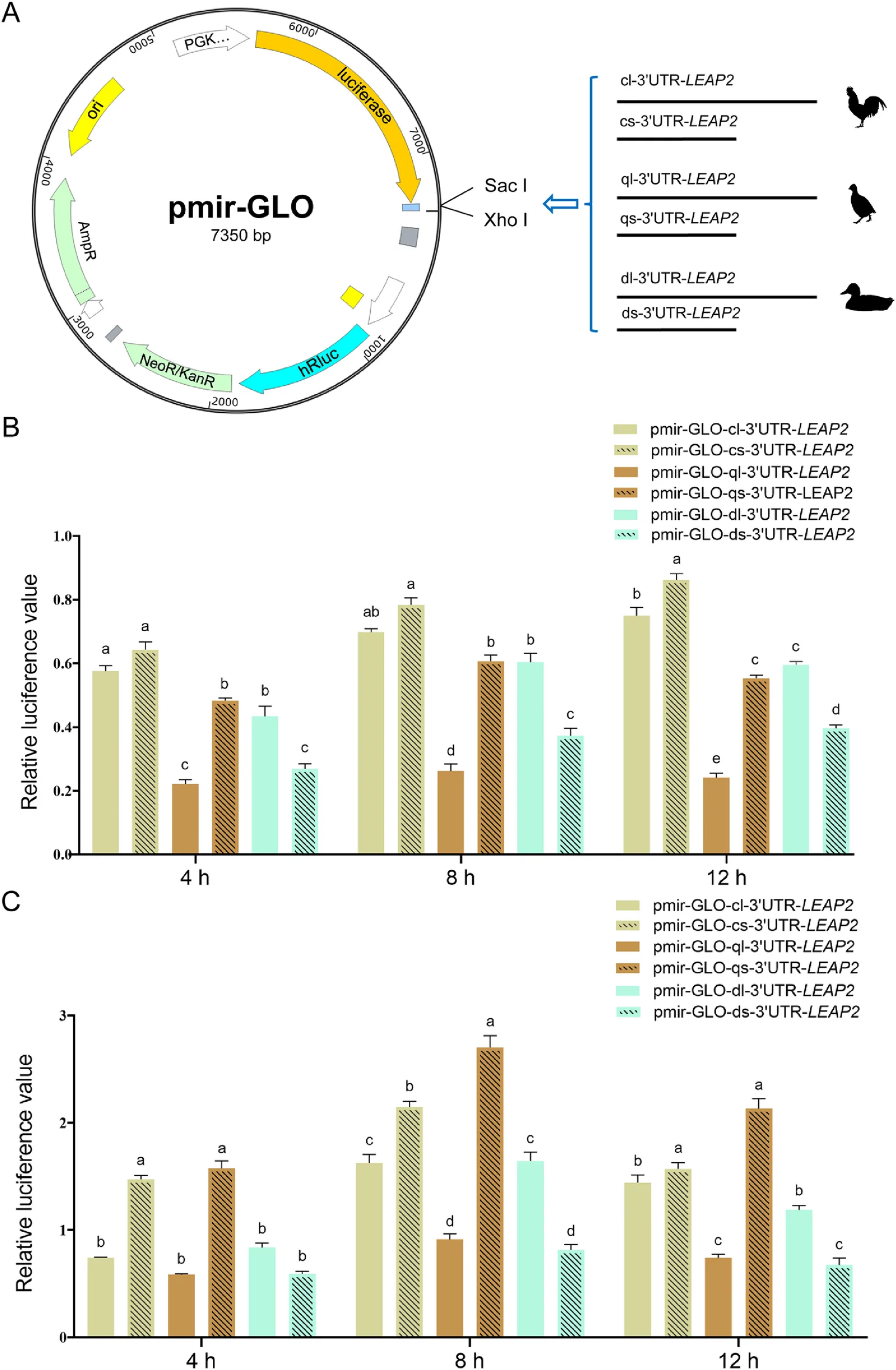
Comparison of stability among different 3′UTRs of the avian *LEAP2* gene. (A) Construction of pmir-GLO vectors containing different 3′UTR variants of the avian *LEAP2* gene. (B) Stability assessment of transfected plasmids in primary chicken embryonic hepatocytes at 4, 8, and 12h post-transfection. Silhouettes of vertebrates were available under public domain licenses at PhyloPic (http://phylopic.org/). Different lowercase letters denote significant differences within the same time point (*P* < 0.05).

### Screening and functional validation of RBPs interacting with the LEAP2 3′ UTR

To further uncover the molecular basis underlying differential stability of l- and s-3′UTRs-*LEAP2*, we analyzed RBP binding motifs within these sequences. RBP prediction revealed abundant RBP binding sites in both s- and l-3′UTRs of chicken, quail and duck *LEAP2*, with a greater number of RBPs predicted on l-3′UTRs (Fig. 5). Analysis of s-3′UTRs showed that the chicken s-3′UTR-*LEAP2* contains multiple stability-promoting RBPs (RBFOX1, SFRS9), whose binding sites are located near the 3′terminus. At the corresponding position, quail possesses one stability-enhancing RBP (SFRS9), whereas neither quail nor duck harbors an RBFOX1 binding site. In addition, both quail and duck have a binding site for a destabilizing RBP near the 3′ end of the s-3′UTR, which is absent at the equivalent position in chicken. The unique extended regions present only in avian l-3′UTRs exhibited high UA content (chicken: 78.95%; quail: 79.75%; duck: 80.82%). RBP prediction for these extended sequences identified one stability-promoting RBP (PABPC1) and one destabilizing RBP (PUM2) in chicken. In quail, two binding sites for the stabilizing RBP ELAVL2 and two sites for destabilizing RBPs (PUM2 and ZFP36) were detected. Only one binding site for the stabilizing RBP ELAVL2 was predicted in duck. RT‑PCR confirmed that all candidate stabilizing and destabilizing RBPs were expressed in primary chicken embryonic hepatocytes and liver tissues, whereas two RBPs (*RBFOX1* and *ELAVL2*) were undetectable in intestinal epithelial cells (Fig. S2). We next constructed pmir-GLO reporter vectors carrying mutations at predicted chicken RBP binding sites. Dual-luciferase assays in primary chicken embryonic hepatocytes showed that gain of PUM2 binding sites in the s-3′UTR-*LEAP2* significantly elevated luciferase activity relative to the cs control. Mutagenesis of RBFOX1 binding sites exerted no significant effect, whereas mutagenesis of SFRS9 binding sites markedly increased luciferase activity (Fig. 6A). These observations suggested that the regulatory effects of these RBPs on short transcript stability were inconsistent with bioinformatic predictions. For the l-3′UTR, mutagenesis of PUM2 and PABPC1 binding sites significantly increased luciferase activity, whereas gain of ZFP36 binding sites reduced luciferase activity(Fig. 6B). Parallel dual-luciferase experiments using primary chicken embryonic intestinal cells demonstrated distinct outcomes. In the s-3′UTR, gain of PUM2 binding sites, or mutagenesis of RBFOX1 or SFRS9 binding sites caused no significant changes in luciferase activity compared with the control (Fig. 6C). In the l-3′UTR, mutagenesis of PUM2 or PABPC1 binding sites, or gain of ZFP36 binding sites also failed to induce significant alterations in reporter activity (Fig. 6D).

**Fig. 5:**
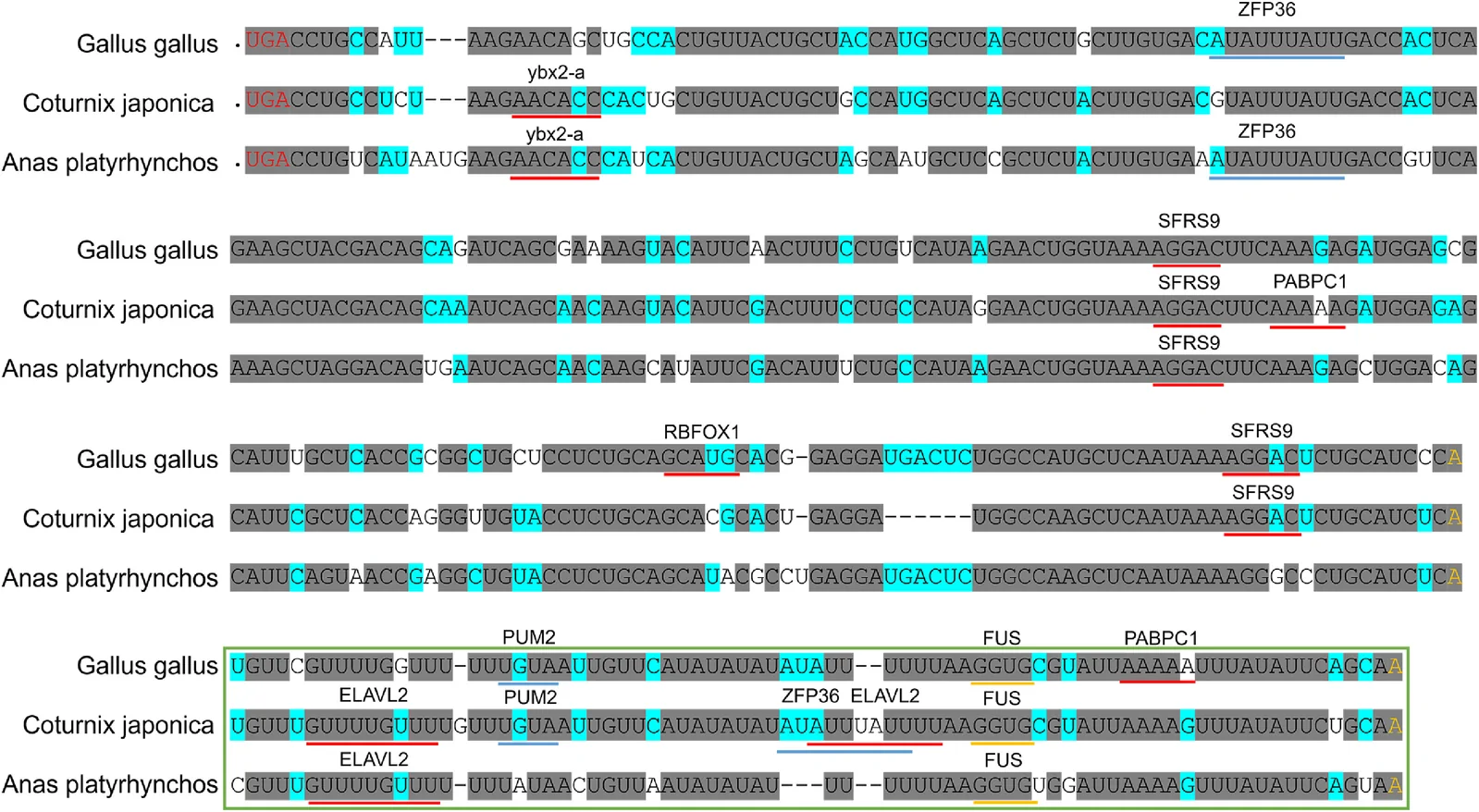
Prediction of RNA-binding proteins (RBPs) in the 3′UTR of the *LEAP2* in chicken, quail, and duck. The prediction of RBPs is achieved using the RBPDB version 1.3.1 (http://rbpdb.ccbr.utoronto.ca/) software. Red underlining indicates stabilizing RNA-binding proteins; green underlining marks destabilizing ones. Yellow underlines indicate RBPs with unknown stabilizing or destabilizing functions. The region highlighted with a green border represents the long transcript of the *LEAP2* gene. Conserved residues across all species are shown in grey, while those conserved in two poultry species are indicated in blue-green.

**Fig. 6:**
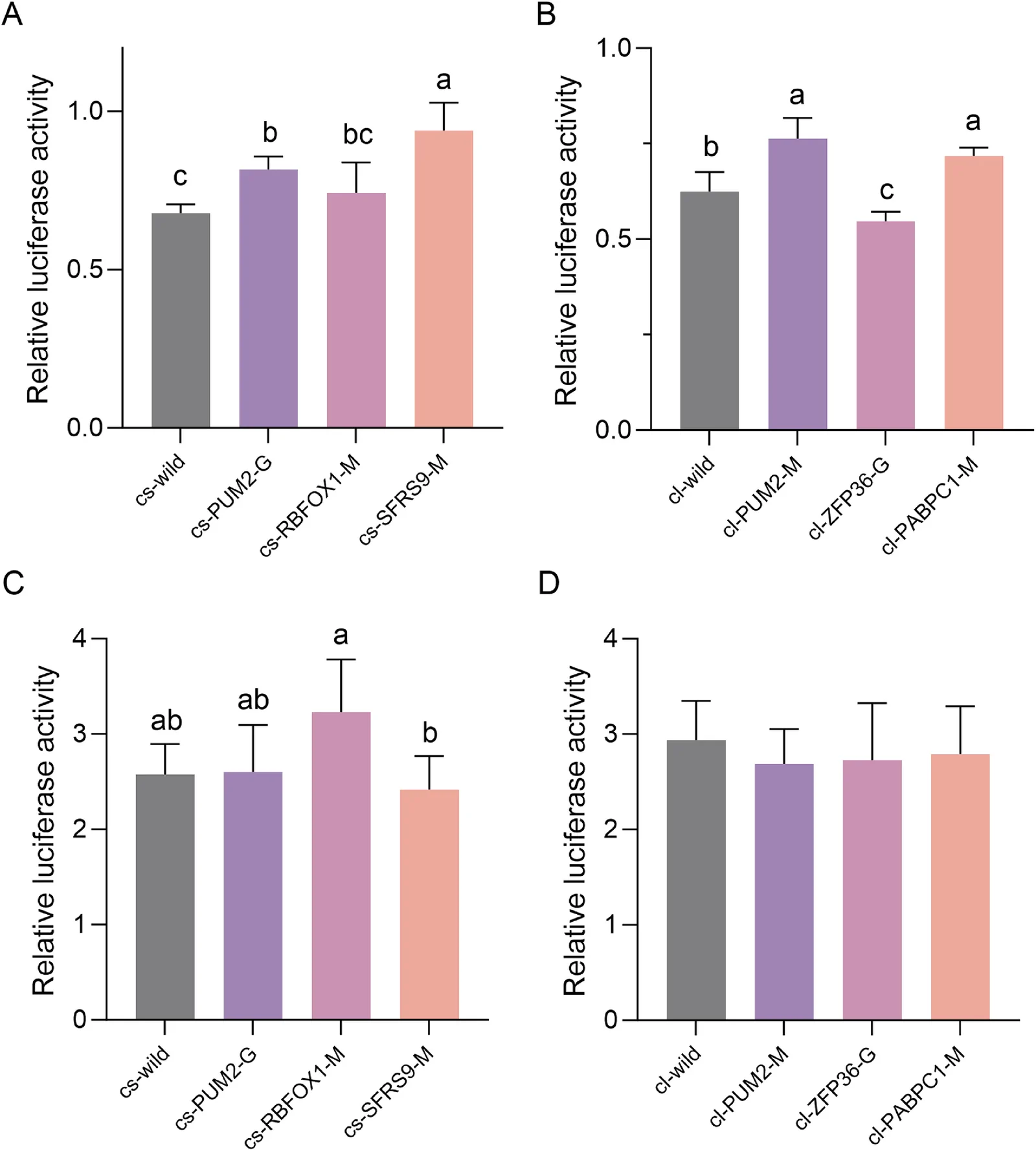
Verification of RBPs binding to the 3′UTR of *LEAP2*. (A, B) Dual-luciferase reporter assays of mutant plasmids in primary chicken embryonic hepatocytes. (C, D) Dual-luciferase reporter assays of mutant plasmids in primary chicken embryonic intestinal cells. Cs, chicken short isoform; Cl, chicken long isoform. -G, gained binding site; -M, mutagenized binding site. Different lowercase letters denote significant differences (*P* < 0.05).

### Screening of target miRNAs binding to the LEAP2 3′ UTR

To further investigate whether miRNAs modulate the stability of the *LEAP2* 3′UTR, we predicted miRNA binding sites within these UTR sequences. Prediction results (Table S7) revealed that the l-3′UTRs-*LEAP2* of chicken and quail contained more miRNA binding sites than their corresponding short isoforms (chicken: 9 vs 4; quail: 11 vs 5). Predicted miRNAs targeting the s-3′UTR-*LEAP2* all exhibited relatively low scores (all below 80). In ducks, the number of predicted miRNA binding sites on the long 3′UTR was comparable to that on the short isoform (14 vs 10), among which three sites had prediction scores higher than 80. Two miRNAs (gga‑miR‑2188‑3p and gga‑miR‑190a‑3p) targeting the chicken l-3′UTR-*LEAP2* were selected for experimental validation. Overexpression of each miRNA in primary chicken embryonic hepatocytes significantly suppressed the expression of l-*LEAP2* and t-*LEAP2* transcripts (Fig. 7A and B). Consistently, inhibitor treatment upregulated the levels of l-*LEAP2* and t-*LEAP2* transcripts (Fig. 7C and D). Collectively, these data indicate that miRNAs serve as one set of regulators governing *LEAP2* transcript stability.

**Fig. 7:**
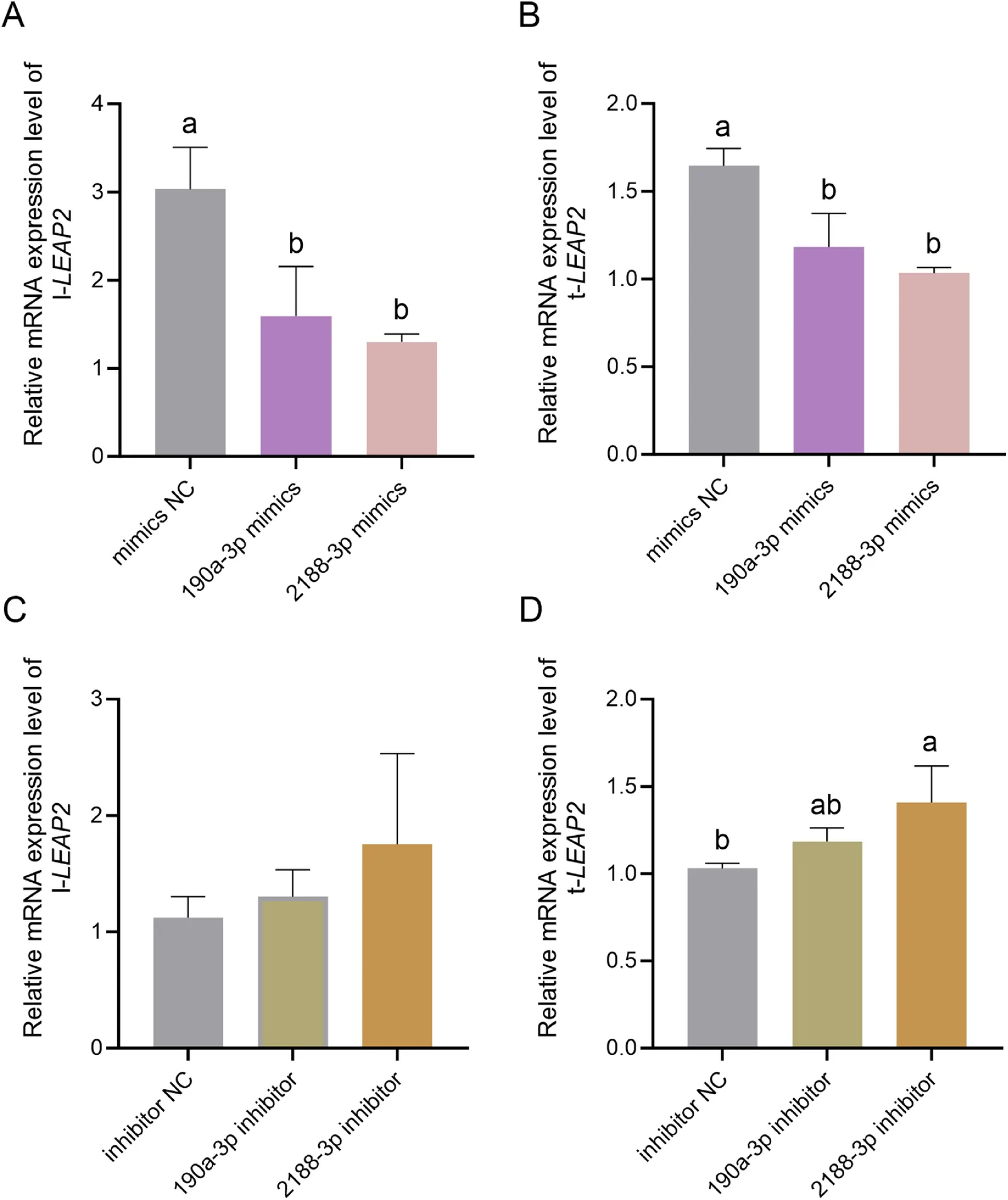
Functional effects of miRNAs on the expression of *LEAP2*. (A, B) Relative mRNA expression levels of l-*LEAP2* and t-*LEAP2* after overexpression of gga-miR-190a-3p and gga-miR-12188-3p in primary chicken embryonic hepatocytes. (C, D) Relative mRNA expression levels of l-*LEAP2* and t-*LEAP2* after inhibition of gga-miR-190a-3p and gga-miR-12188-3p in primary chicken embryonic hepatocytes. Different lowercase letters denote significant differences (*P* < 0.05).

## DIscussion

LEAP2 is a recently identified liver-intestine-enriched peptide regulating appetite and metabolism (Ge et al., 2018). It has attracted substantial research attention in human clinical investigations (Mani et al., 2019; Hagemann et al., 2022; Bhargava et al., 2023) and poultry studies (Zheng et al., 2022; Shu et al., 2024; Saffar et al., 2025; Chen et al., 2026). Nevertheless, few studies have addressed the transcriptional regulation of the *LEAP2* gene, particularly investigations concerning its transcripts. Cloning the mRNA sequence of a gene requires clarification of its primary expression tissues. Tissue profiling analyses of *LEAP2* in chickens and quails have revealed predominant expression in the liver and intestine. However, the expression pattern of *LEAP2* in ducks, a representative waterfowl species, remains poorly characterized. Moreover, the expression dynamics of *LEAP2* under distinct energy statuses are still unclear. Accordingly, the present study first characterized the tissue expression profiles of *LEAP2* in ducks and mice. Duck *LEAP2* exhibited high expression in the liver, kidney, duodenum, jejunum and ileum, consistent with findings in quails (Shu et al., 2024), indicating a conserved tissue expression pattern of *LEAP2* across avian lineages. By contrast, chicken *LEAP2* shows relatively low expression in the kidney (Zheng et al., 2022). This interspecies discrepancy within poultry may be explained by divergent renal physiological adaptations. Mouse *Leap2* is also predominantly expressed in the liver and small intestine, demonstrating that the core tissue localization of *LEAP2* in key metabolic organs is evolutionarily conserved between avian and mammalian species.

Based on Animal‑APAdb database analysis, the chicken *LEAP2* gene harbors two distinct polyadenylation site clusters within its 3′UTR (Table S8), indicating active APA events of *LEAP2* in chicken. In the present study, 5′/3′‑RACE assays identified two *LEAP2* transcripts in poultry and mice that share identical coding sequences but differ in the length of their 3′UTRs, which represents a typical APA event. This phenomenon appears to be conserved between poultry and mice, yet additional evidence is required to verify whether such conservation extends to other mammals. The chicken s-*LEAP2* transcript cloned herein is largely consistent with the reference sequence deposited in NCBI, with only minor variations including several SNPs and a single-base deletion. The chicken l-*LEAP2* transcript identified in this work represents a previously unreported isoform. For duck *LEAP2*, the long transcript contains a 3′UTR matching the reference genome annotation; however, both cloned duck transcripts possess shorter 5′UTRs relative to the reference sequence. This observation may explain the earlier finding from multi-species sequence alignment that duck *LEAP2* exhibits an apparently extended 5′coding region (i.e., longer signal peptide) compared with orthologs from other species (Shu et al., 2024), which likely originates from erroneous database annotation. Similar annotation discrepancies may also exist in other species such as ostriches. We therefore propose that *LEAP2* transcripts should be characterized in a broader range of species. Such efforts will facilitate correction of existing reference genome annotations and enable systematic elucidation of the regulatory patterns governing *LEAP2* transcript expression across vertebrates.

Sequence alignment using DNAMAN revealed high sequence similarity of the l-*LEAP2* transcripts among avian species (97% identity for s-UTRs and 98% identity for l-UTRs). In contrast, the l- and s-UTR sequences of mouse *Leap2* exhibit substantial divergence from their avian counterparts, yielding poor alignment results. These findings indicate that the *LEAP2* genomic sequences of mammals have diverged considerably from those of avians during long-term evolution, whereas the mature peptide-coding sequence of LEAP2 remains highly conserved across vertebrates (Li et al., 2021; Shu et al., 2024). The full-length mouse *Leap2* transcripts cloned in this study provide a foundation for future investigations into the transcriptional regulation and distinct physiological functions of individual *LEAP2* transcript isoforms at the genetic level.

When examining gene expression under divergent feeding conditions across species, we observed that the t-*LEAP2* transcripts and l-*LEAP2* transcripts in tissues such as the liver and intestine undergo dynamic changes corresponding to varying energy statuses in chickens (including AA broiler chicks and 42-day-old AA broilers), ducks and quails. This observation is consistent with previous findings in quails (Shu et al., 2024). Nevertheless, in mouse models subjected to different feeding regimes, *Leap2* expression in its major expression tissues did not display significant alterations upon changes in feeding status (Fig. 3C). Earlier work demonstrated that provision of casein (a dietary protein component) alone failed to induce significant changes in *Leap2* expression in the duodenum and jejunum of mice. After administration of a mixed diet, only a mild, statistically non-significant upward trend of *Leap2* was detected in the mouse small intestine, which aligns with our present results. By contrast, *LEAP2* expression was markedly elevated in human jejunal organoids stimulated with glucose, fatty acids and insulin in combination (Johansen et al., 2025). The divergent responsiveness of *LEAP2* expression to dietary cues between mice, humans and poultry suggests interspecies differences in the intestinal *LEAP2* response to diet-hormone signaling. Furthermore, although two *LEAP2* transcript isoforms exist in all tested avian species, the proportion of the l-*LEAP2* transcript following feeding varies markedly across species. In the refed group, the proportion of l-*LEAP2* transcripts relative to t-*LEAP2* transcripts was higher in ducks than in chickens and quails; this pattern was even more pronounced in mice, in which nearly all detected transcripts corresponded to the long isoform after starvation and refeeding (Fig. 3). In the fed group (ad libitum feeding), however, the proportional abundance of l-*LEAP2* transcripts in ducks was lower than that in chickens and quails. We hypothesize that such differential abundance of short and long transcript isoforms may be linked to mRNA stability, which directly governs protein translation efficiency (Wu and Bazzini, 2023; Dowdle and Lykke-Andersen, 2025). Given the pivotal role of *LEAP2* in energy homeostasis, further research is warranted to characterize how distinct 3′UTR sequences modulate *LEAP2* mRNA stability.

Given that both *LEAP2* transcript isoforms in avians respond to variations in feed intake, whereas mouse *Leap2* transcripts are unaffected by feeding status, we further experimentally investigated the stability of avian *LEAP2* 3′UTRs. In this study, pmir-GLO luciferase reporter vectors carrying distinct avian *LEAP2* 3′UTR sequences were constructed. Luciferase activity measurement was performed to indirectly evaluate the stability and translational efficiency of different UTRs. The results revealed that the s-3′UTR of *LEAP2* exhibited higher stability than the l-3′UTR in chickens and quails. Conversely, the l-3′UTR of duck *LEAP2* was more stable than its short counterpart. These data indicate that divergent UTR sequences possess distinct stabilities, which consequently lead to varied translational efficiencies. This mechanism may account for the differential abundance of *LEAP2* transcript isoforms across avian species. Following feeding, the l-*LEAP2* transcript is more stable in ducks and thus accumulates at higher levels, while the short transcript harbors a more stable UTR in chickens and quails and shows relatively greater abundance. Such observations are consistent with established cell biological principles.

Predicted RBPs binding profiles within the *LEAP2* 3′UTR can generally explain the molecular mechanisms underlying the higher stability of s-*LEAP2* transcripts relative to long isoforms in chickens and quails, as well as the greater stability of the l-3′UTR-*LEAP2* compared with the s-3′UTR in ducks. During cellular experimental validation, the functional outcomes of several RBPs detected for the s-3′UTR deviated from predictions or even displayed opposite effects. This discrepancy may be attributed to the dependence of RBP binding on surrounding UA-rich sequences. Although RBPs are present and capable of binding target regions, flanking UA sequences can modulate their regulatory functions. Two RBPs located in the l-3′UTR were validated in hepatocytes, and these RBPs exerted pronounced regulatory effects on the l-3′UTR. Nevertheless, the result for PABPC1 contradicted our expectation. Collectively, these data demonstrate that RBPs constitute one of the regulators governing long UTR stability, yet they are not the sole determinant. The inconsistent results observed in intestinal cells warrant further experimental verification in future investigations.

Apart from RBP binding to the 3′UTR, additional factors modulating mRNA stability via the 3′UTR include m⁶A modification, miRNAs, A-to-I editing, lncRNAs and circRNAs (Wang et al., 2015; Navarro et al., 2021) . Our prediction analysis revealed that the l-3′UTRs-*LEAP2* of chickens and quails harbor more miRNA binding sites than their short UTR counterparts, whereas the number of miRNA binding sites in the duck long UTR is roughly comparable to that in the short UTR. Two miRNAs (gga‑miR‑2188‑3p and gga‑miR‑190a‑3p) targeting sequences within the long UTR were selected for experimental validation. Both miRNAs exhibited moderate expression levels in primary chicken embryonic hepatocytes, with gga-miR-2188 showing relatively higher abundance. Overexpression of either miRNA significantly suppressed the expression of the l-*LEAP2* transcript and t-*LEAP2* transcripts in primary chicken embryonic hepatocytes. Conversely, inhibition of these miRNAs via corresponding inhibitors elevated the levels of the l-*LEAP2* transcript and t-*LEAP2* transcripts. These results confirm that miRNAs serve as one set of regulators controlling *LEAP2* mRNA stability.

In conclusion, this study identifies two conserved *LEAP2* isoforms generated by APA that differ in 3′UTR length across avian species and mice. Both isoforms are mainly expressed in liver and intestine and responsive to feeding status. The l‑3′UTR isoform accounts for two‑thirds of t-*LEAP2* transcripts in refed ducks. 3′UTR‑dependent transcript stability exhibits species‑specific patterns in poultry: the s‑3′UTR is more stable in chicken and quail, while l‑3′UTR is more stable in duck. RBPs and miRNAs jointly modulate *LEAP2* 3′UTR stability, though additional regulators remain to be identified. Collectively, these findings reveal species‑specific regulatory patterns of *LEAP2* isoforms in avians in response to feeding fluctuations, which may be associated with feed intake and energy metabolism. The precise physiological functions of these isoforms require further investigation.

## Funding

This work was supported by the 10.13039/501100001809National Natural Science Foundation of China (32302725), the 10.13039/501100004608Natural Science Foundation of Jiangsu Province (BK20220648), and the Postgraduate Research and Practice Innovation Program of Jiangsu Province (26CXJH7495).

## Author contributions

Hui Wang and Xin Shu made substantial contributions to the acquisition and analysis of experimental data, performed the majority of laboratory experiments, and drafted the original manuscript. Yutong Wang, Bingjie Xu, and Li Liu contributed to sample collection and RT-qPCR data acquisition and analysis, and critically reviewed the manuscript. Yimeng Li and Ziqiu Niu performed bioinformatic analysis and result interpretation, and critically reviewed the manuscript. Xiaotong Zheng conceived and designed the study and critically reviewed the manuscript. Jianfei Chen guided the experimental framework, interpreted results, revised the manuscript critically, and supervised the study. All authors have read and approved the final version of the manuscript and agree to be accountable for all aspects of the work.

## Disclosures

The authors declare that they have no known competing financial interests or personal relationships that could have appeared to influence the work reported in this paper.

## Footnotes

Supplementary material associated with this article can be found, in the online version, at doi:10.1016/j.psj.2026.108027.
