# Supplementary material for: Characterization of avian LEAP2 transcripts with alternative polyadenylation and their 3′UTR stability
Source: Poult Sci. 2026 Oct 21;105(12):108027. doi: 10.1016/j.psj.2026.108027 (PMC13734868; doi:10.1016/j.psj.2026.108027)
Supplement: Supplementary file 1 [file mmc1.docx]

**SUPPLEMENTARY DATA**

**Fig. S1.** Relative expression of *LEAP2* isoforms in the main tissues of adult AA broiler chicken. The "t" and "l" in t-*LEAP2* and l-*LEAP2* stand for "total" and "long", respectively. Different lowercase letters denote significant differences within the same tissue (*P* < 0.05).

**Fig. S2.** RT-PCR gel images of several important predicted RNA-binding proteins that can bind to the 3′UTR sequence of the *LEAP2* gene in chicken liver tissue (A) and chicken embryonic hepatocytes (B).
